# Transcriptomic comparison of ovarian granulosa cells between adult sheep and prepubertal lambs

**DOI:** 10.1186/s12864-022-08379-x

**Published:** 2022-02-21

**Authors:** Hao Tian, Panyu Ren, Kailing Liu, Chunjuan Qiu, Lihong Fan, Junlong Li, Jian Hou

**Affiliations:** 1 State Key Laboratory of Agrobiotechnology and College of Biological Science, China Agricultural University, Beijing, China; 2 Inner Mongolia, Sino Sheep Breeding Co. Ltd, Wulanchabu, Inner Mongolia China

**Keywords:** RNA-seq, Transcriptome, Granulosa cells, Sheep, Prepubertal lamb

## Abstract

**Background:**

The oocyte development ability of prepubertal animals is significantly lower than that of adult animals. Granulosa cells (GCs) have an important function on regulation of follicular and oocyte development. Therefore, analysis of GC characteristics can be used to explore the developmental mechanism of follicles and oocytes.

**Results:**

In order to understand the possible reasons for the differences in follicle and oocyte development between lambs and adult sheep, we utilized high-throughput sequencing technique to analyze the transcriptome of GCs from follicle-stimulating hormone (FSH) superstimulated adult ewes and prepubertal lambs. Adult ewes were treated with FSH for 3 days (group A) and lambs were FSH-treated for 2 days (group B) or 3 days (group C). Transcriptome analysis of GCs showed that there were 405 and 159 differentially expressed genes from A vs. B and A vs. C, respectively. The results indicated that prolonging the FSH-treatment of lambs made the GC state of lambs more similar to the adult ewes, but there were still a large number of differentially expressed genes between adult ewes and lambs. Further analysis showed that many differently expressed genes were implicated in cell proliferation and apoptosis, oocyte development and follicular ovulation. Cellular examination demonstrated that fatty acid binding protein 4 (*FABP4*), which was highly expressed in lamb GCs, had a potential of promoting cell apoptosis. Cytoplasmic phospholipase A2 (*PLA2G4A*), which was expressed lowly in lamb GCs, may be responsible for reduced synthesis of prostaglandins in cells and impaired follicle/oocyte development. In contrast, glutathione S-transferase β-1 (*GSTT2B*) and forkhead boxO6 (*FOXO6*) had no apparent effect on the proliferation and apoptosis of GCs.

**Conclusions:**

Our study found dramatic transcriptomic differences in GCs between lambs and adult sheep, which may explain the possible reasons for the defects of follicle and oocyte development in lambs compared to adult sheep. Our data provides important information for further understanding the mechanism of follicular development in prepubertal animals and improving their oocyte developmental competence.

**Supplementary Information:**

The online version contains supplementary material available at 10.1186/s12864-022-08379-x.

## Background

The technology of in vitro embryo production with juvenile animals as oocyte donors, so called juvenile in vitro embryo transfer (JIVET), has a great application prospect in livestock breeding [1]. However, the embryonic development ability of oocytes from prepubertal animals is quite low and thus limits the application of JIVET technology in large scale [2, 3]. In sheep, gonadotrophin treatment of prepubertal lambs can stimulate a great number of follicles to grow up to medium sizes (2–4 mm in diameter) at oocyte aspiration, but the derived oocytes are always not competent during subsequent development in vitro when used in JIVET program [4, 5]. The reasons for poor development of prepubertal oocytes are not fully understood and remains to be investigated.

In vivo, the oocytes grow and develop in follicles and suboptimal follicular environment may compromise the oocyte competence. Within an antral follicle, the oocyte is exposed to follicular fluid (FF) and enclosed by surrounding granulosa cells (GCs). GCs exert very important roles on oocyte development through secretion of factors into FF or direct communication with oocytes via gap junctions [6, 7]. Thus, the activity of GCs is correlated with oocyte developmental potential.

Recently, transcriptomic and proteomic analysis of GCs found that hundreds of genes or proteins were differentially expressed between adult ewes and juvenile lambs [8, 9]. Proteomic profiling identified lots of proteins differentially abundant in lamb FF compared to adult ewes [10]. Transcriptional analysis of whole ovaries revealed that the expression patterns of miRNAs and mRNAs were quite different between adults and lambs [11]. Similarly in bovine, prepubertal heifers and adult cows had distinct properties of follicular environment and many genes that influence follicular functions and oocyte development were differently expressed in GCs between heifers and cows [12, 13]. These studies suggest that the functional insufficiency of prepubertal follicular environment may explain the reduced oocyte quality of prepubertal animals. However, previous studies have not strictly correlated the developmental ability of oocytes with the gene expression of GCs. In addition, the possible functions of identified genes need to be tested in cells.

To further understand the mechanism of prepubertal follicle development, in the present study we compared the transcriptome of GCs between lambs and adults under the same superovulation regime and validated the effects of some differently expressed genes. Based on the data, we indicate that FSH-treatment of lambs for 3 days is more favorable to follicle and oocyte development than treatment for 2 days, but there are still significant differences in GC function between lambs and adult ewes.

## Results

### Development ability of adult and lamb oocytes

Table 1 shows the efficiency of oocyte harvest and embryo production from adult and prepubertal lamb donors. After FSH-treatment, the average number of oocytes recovered in the two lamb groups was much higher than that of adult ewes. After in vitro maturation and fertilization, there was no significant difference in cleavage rate between lambs and adults, but the blastocyst rate in the adult group was significantly higher than that of both lamb groups. There was no significant difference in the cleavage rate between the two lamb groups, but the blastocyst rate in the 3-day FSH-treatment group was significantly higher than that in the 2-day FSH-treatment group (*p* < 0.05).

**Table 1:** Developmental competence of adult and lamb oocytes

Groups	No. of donors	No. of recovered oocytes	Average no. of oocytes recovered from each donor	No. of cleaved (Mean ± SEM, %)	No. of blastocysts (Mean ± SEM, %)
Adult	100	1202	12.2	823 (68.4 ± 4.9)	83/199 (40.5 ± 1.9) ^a^
Lamb-2D	5	635	127	489 (76.2 ± 3.5)	59/363 (17.8 ± 1.4) ^b^
Lamb-3D	5	560	112	450 (78.2 ± 9.1)	67/255 (27.9 ± 3.1) ^c^

Percentage of cleaved: No. of cleaved /No. of recovered oocytes

Percentage of blastocysts: No. of blastocysts/No. of cleaved. Only parts of embryos were cultured for evaluation of their development

Values with different letters (a, b and c) within the same column are significantly different (*p* < 0.05)

*Adult* Adult ewes were treated with FSH for 3 days, *Lamb-2D* Lambs were FSH-treated for 2 days, *Lamb-3D* lambs were FSH-treated for 3 days

### Global gene expression

**Fig. 1:**
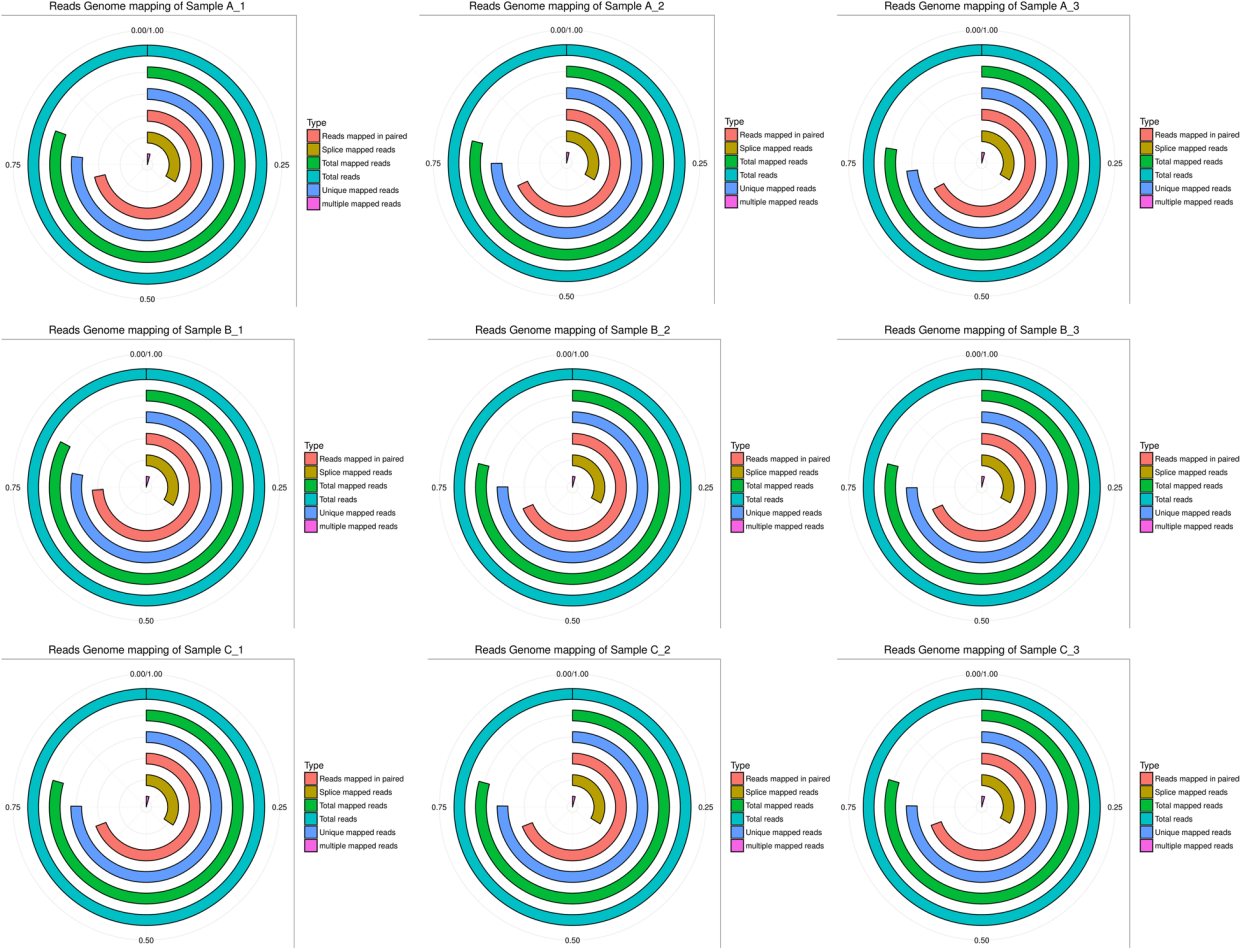
The rate of reads mapped onto the genome. Each loop represents one alignment type and the radian of loop represents the rate of alignment. The total reads represent total clean reads of samples, and the ratio is 1. A_1-A_3: adult ewe group; B_1-B_3: group of lambs with 2-day FSH-treatment; C_1-C_3: group of lambs with 3-day FSH-treatment

The transcriptome of ovine GCs was analyzed by global RNA sequencing of each sample. After filtered out low-quality reads and removed the adaptor sequences, we finally obtained 98.36–99.4% clean data in each sample (Supplemental Table S1). As shown in Fig. 1, we calculated the rate of all reads from each sample mapped onto the genome, and showed that 73.30%-77.95% of the reads were uniquely mapped onto the ovine genome (https://www.ncbi.nlm.nih.gov/genome/?term=Ovis%20Aries). These data were used to analysis of gene expression.

### Differentially expressed genes (DEGs)

**Fig. 2:**
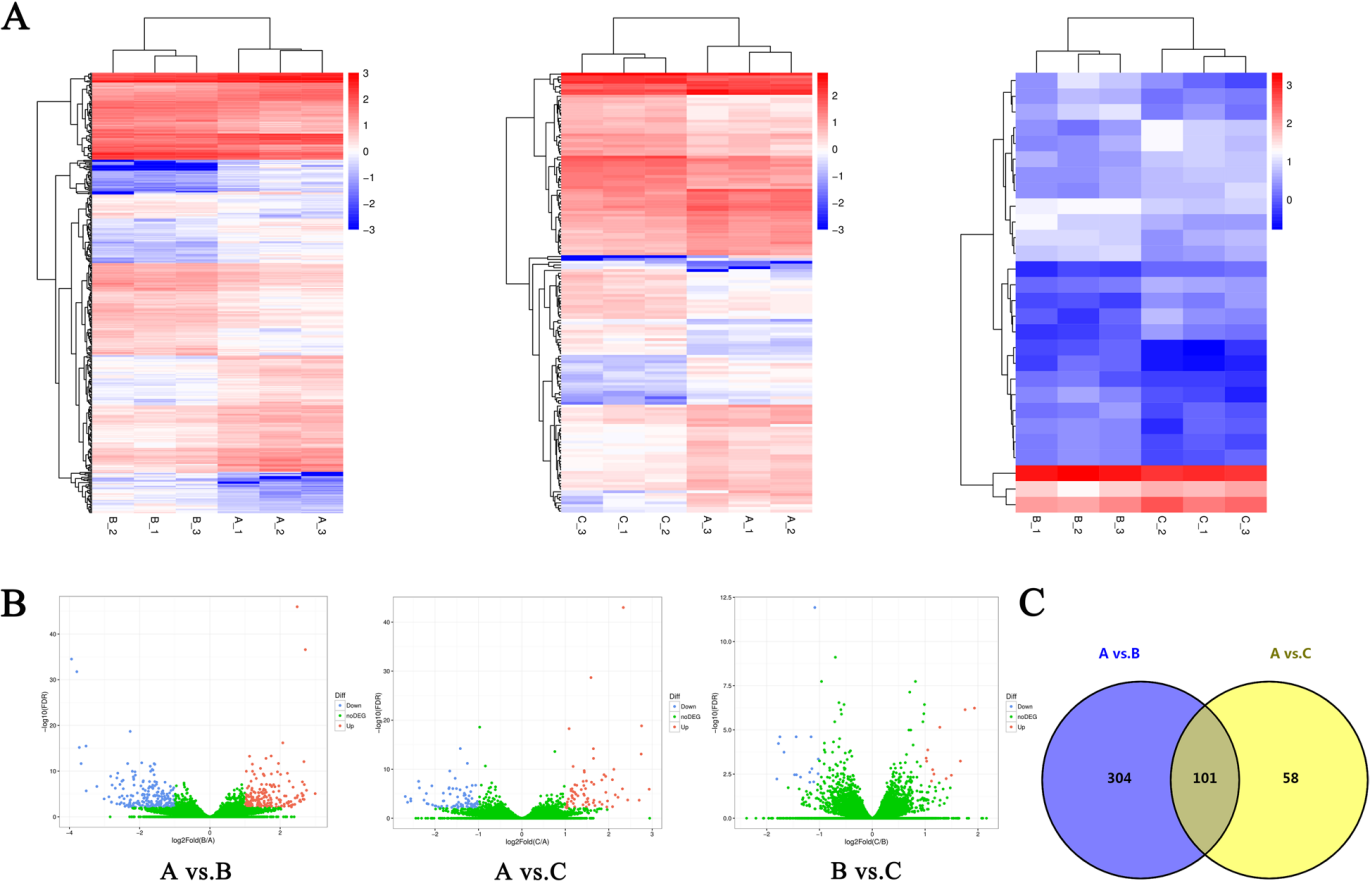
Cluster and filter analysis of DEGs. **A** Heatmaps of the differentially expressed genes (DEGs). The color key from blue to red indicates the relative gene expression level from low to high, respectively. **B** Volcano plots show DEGs. The x-axis shows the fold-change in gene expression, and the y-axis shows significant statistical differences. Red, up-regulated genes; blue, down-regulated genes; green, genes with no significant difference in expression. **C** Venn diagram shows common DEGs between pair-wise comparison. A vs. B: comparison between adult ewes and lambs with 2-day FSH-treatment; A vs. C: comparison between adult ewes and lambs with 3-day FSH-treatment; B vs. C: comparison between lambs with 2-day FSH-treatment and with 3-day FSH-treatment

Differentially expressed genes between groups were identified using criteria including q-value ≤ 0.01 and |log2FC |> 1. The expression profiles of DEGs are shown in Fig. 2A and B and detailed in supplementary Table S2. We found 405 DEGs between adult ewes (A) and lambs with 2-day FSH-treatment (B) (referred to as A vs. B), and the number of up- and down-regulated genes was 190 and 215 in B group, respectively. There were 159 DEGs between adult ewes and lambs with 3-day FSH-treatment (C) (referred to as A vs. C), including 72 up- and 87 down-regulated genes in C group, respectively. For B vs. C, 28 DEGs were found, of which 12 were up-regulated and 16 were down-regulated in C group. Overall, the number of DEGs from A vs. C was decreased by 246 compared to A vs. B.

We further explored the DEGs through pairwise comparative analysis of different groups and found 101 common DEGs existed in A vs. B & A vs. C (Fig. 2C and supplementary Table S2). Among them, 53 genes were up-regulated and 48 genes were down-regulated in both lamb groups compared to adult group.

### Analysis of GO and KEGG pathways

**Fig. 3:**
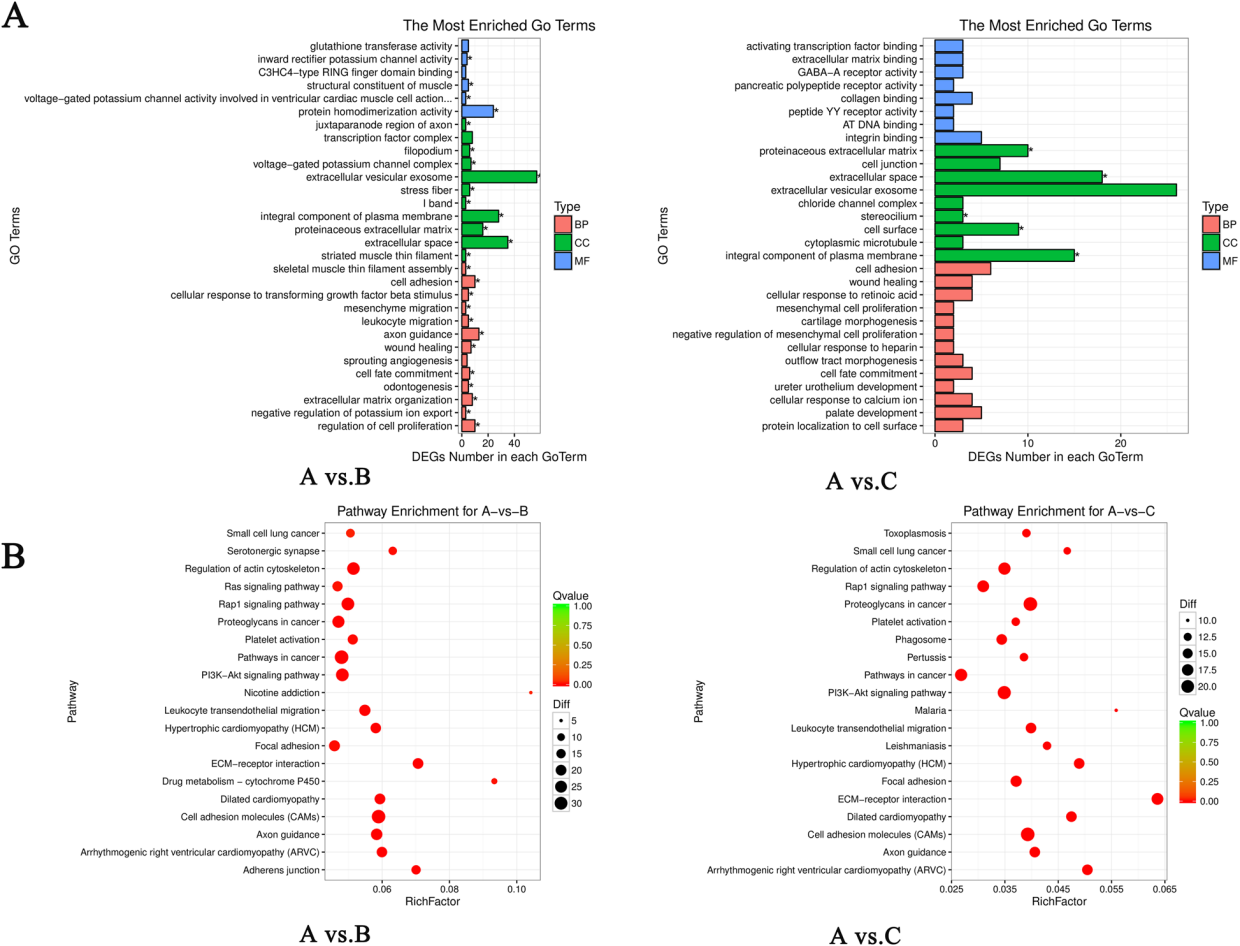
Screening and enrichment analysis of the differentially expressed genes (DEGs). **A** GO enrichment analysis of DEGs. Top 30 significant terms from enrich Gene Ontology for biological processes (BP), cellular components (CC) and molecular functions (MF). **B** KEGG enrichment analyses of DEGs. The ratio of the number of DEGs to the total gene number is represented by the enrichment factor. Size of dots: number of genes; color of dots: range of *p*-values. A vs. B comparison between adult ewes and lambs with 2-day FSH-treatment; A vs. C: comparison between adult ewes and lambs with 3-day FSH-treatment

In order to further investigate the biological relevance of all DEGs, we performed GO analysis of DEGs to identify enrichment of biological processes in each group. The DEGs from A vs. B and A vs. C were enriched in many identical GO terms, but the enriched GO terms from A vs. B were more than A vs. C (2114 vs. 1178). The top 30 GO terms of biological process (BP), cellular component (CC) and molecular function (MF) of A vs. B and A vs. C are shown in Fig. 3A. All DEGs were mapped to the KEGG database to further investigate their functions. A total of 405 DEGs from A vs. B were categorized into 231 pathways, and 159 DEGs from A vs. C were categorized into 181 pathways (q-value ≤ 0.05). The representative top 20 pathways are shown in Fig. 3B.

Next, we conducted GO and KEGG enrichment analysis for the common DEGs from A vs. B & A vs. C. As shown in Table 2 and supplementary Table S3, the common DEGs were enriched in 767 GO terms, including 533 biological processes, 91 cell components and 143 molecular functions. A total of 16 GO terms were enriched significantly (*p* < 0.05), including 9 biological processes (such as cell adhesion, extracellular matrix organization and regulation of cell proliferation), 5 cellular components (such as proteinaceous extracellular matrix, cell surface, and extracellular vesicular exosome), and 2 molecular functions (structural constituent of muscle and protein homodimerization activity). KEGG pathway analysis revealed that the common DEGs were enriched in 160 pathways and significantly enriched in 38 pathways, including adherens junction, regulation of lipolysis in adipocytes, ECM-receptor interaction, PI3K-Akt pathway, and protein digestion and absorption signaling pathway.

**Table 2:** GO/KEGG Enrichment analysis of differentially expressed genes among different comparisons

	A vs. B & A vs. C
	Intersection
Biological process	533 (9)
Cellular component	91 (5)
Molecular function	143 (2)
KEGG Pathway	160 (40)

The value in parenthesis is the significant enrichment number of differentially expressed genes with *p*-value ≤ 0.05

### Protein–Protein Interaction (PPI) network analysis

**Fig. 4:**
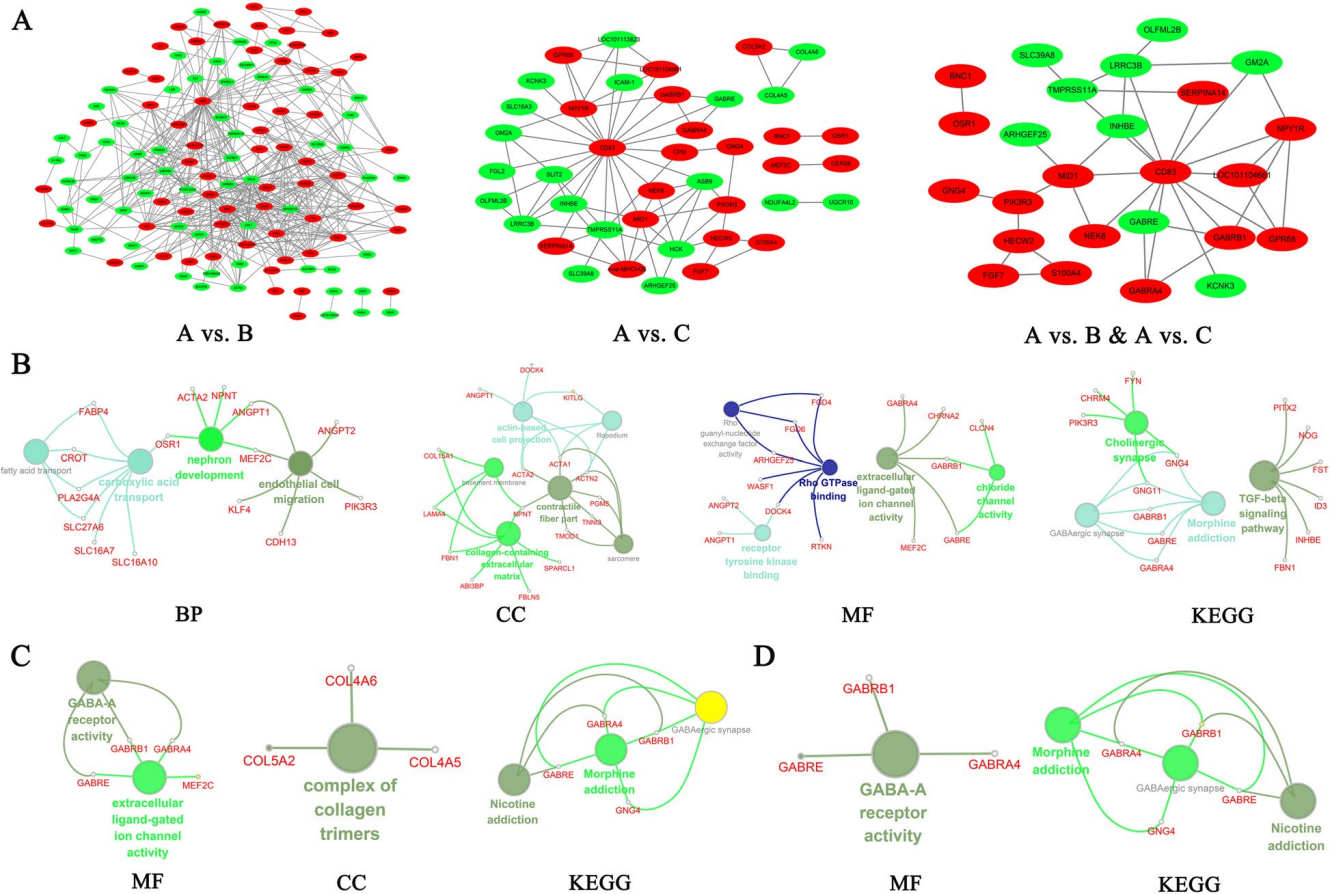
Analysis of protein–protein interaction (PPI) of the DEGs. **A** Integral PPI networks of A vs. B, A vs. C and A vs. B & A vs. C. The red node represents up-regulated genes and green node represents down-regulated genes. **B**-**D** Gene functional annotation of the differentially expressed genes, (**B)** represents A vs. B, (**C**) represents A vs. C, (**D)** represents A vs. B & A vs. C. A vs. B: comparison between adult ewes and lambs with 2-day FSH-treatment; A vs. C: comparison between adult ewes and lambs with 3-day FSH-treatment; B vs. C: comparison between lambs with 2-day FSH-treatment and with 3-day FSH-treatment. A vs. B & A vs. **C**: the common part of A vs. B and A vs. C

When protein-protein interaction of the DEGs were analyzed, we found that the PPI network of A vs. B was reconstructed from 123 gene symbols (Figure 4A and supplementary Table S4). Biological function analysis on the gene symbols by clueGO revealed that the genes were related to endothelial cell migration, nephron development, carboxylic acid transport, fatty acid transport (BP), contractile fiber part, collagen-containing extracellular matrix, actin-based cell projection (CC) and GABA-A receptor activity (MF) (Figure 4B). The genes were involved in TGF-beta signaling, cholinergic synapse and morphine addiction pathways. The PPI network of A vs. C was reconstructed from 41 gene symbols (Figure 4A). The genes were related to complex of collagen trimers (CC), GABA-A receptor activity and extracellular ligand-gated ion channel activity (MF) (Figure 4C). The genes were involved in nicotine addiction GABAergic synapse and morphine addiction pathways. The PPI network of A vs. B & A vs. C was reconstructed from 24 gene symbols (Figure 4A). The genes were related to GABA-A receptor activity (MF) and involved in nicotine addiction, GABAergic synapse and morphine addiction pathways (Figure 4D).

### Validation of expression levels of DEGs

**Fig. 5:**
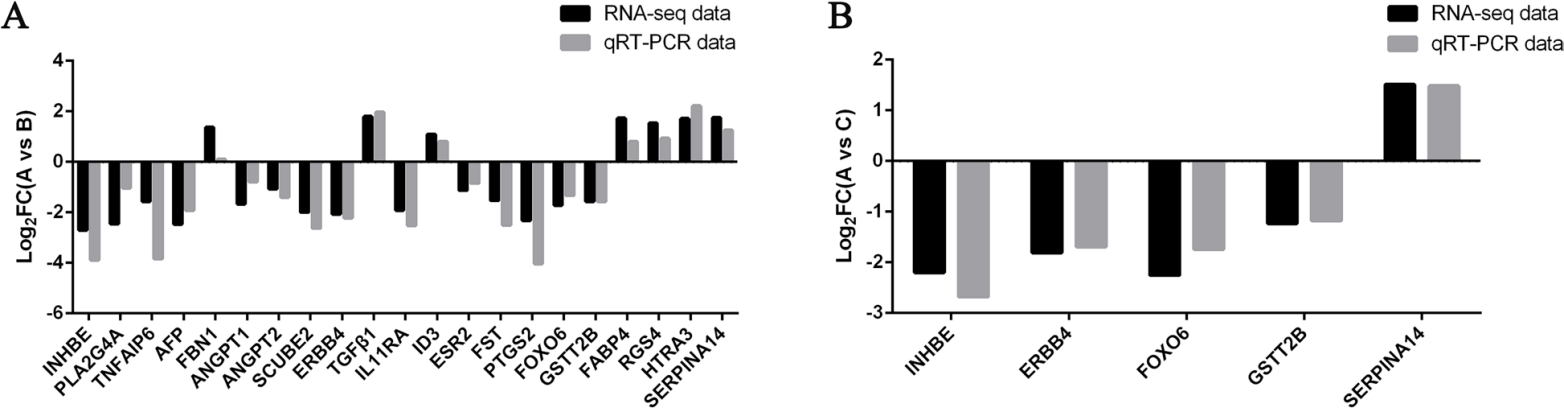
Verification of expression levels of differentially expressed genes by qRT-PCR. **A** Comparison of gene expression between adult ewes and lambs with 2-day FSH-treatment. **B** Comparison of gene expression between adult ewes and lambs with 3-day FSH-treatment

Nineteen genes associated with follicle development, oocyte maturation and ovulation were selected for expression verification by qRT-PCR. The results showed that the trend of up- and down-regulation of gene expression was consistent with the RNA-seq, and the expression was significantly different between adult and lamb GCs (Fig. 5A and B), proving that our data of high-throughput RNA-seq were reliable.

### Functional verification of some genes

**Fig. 6:**
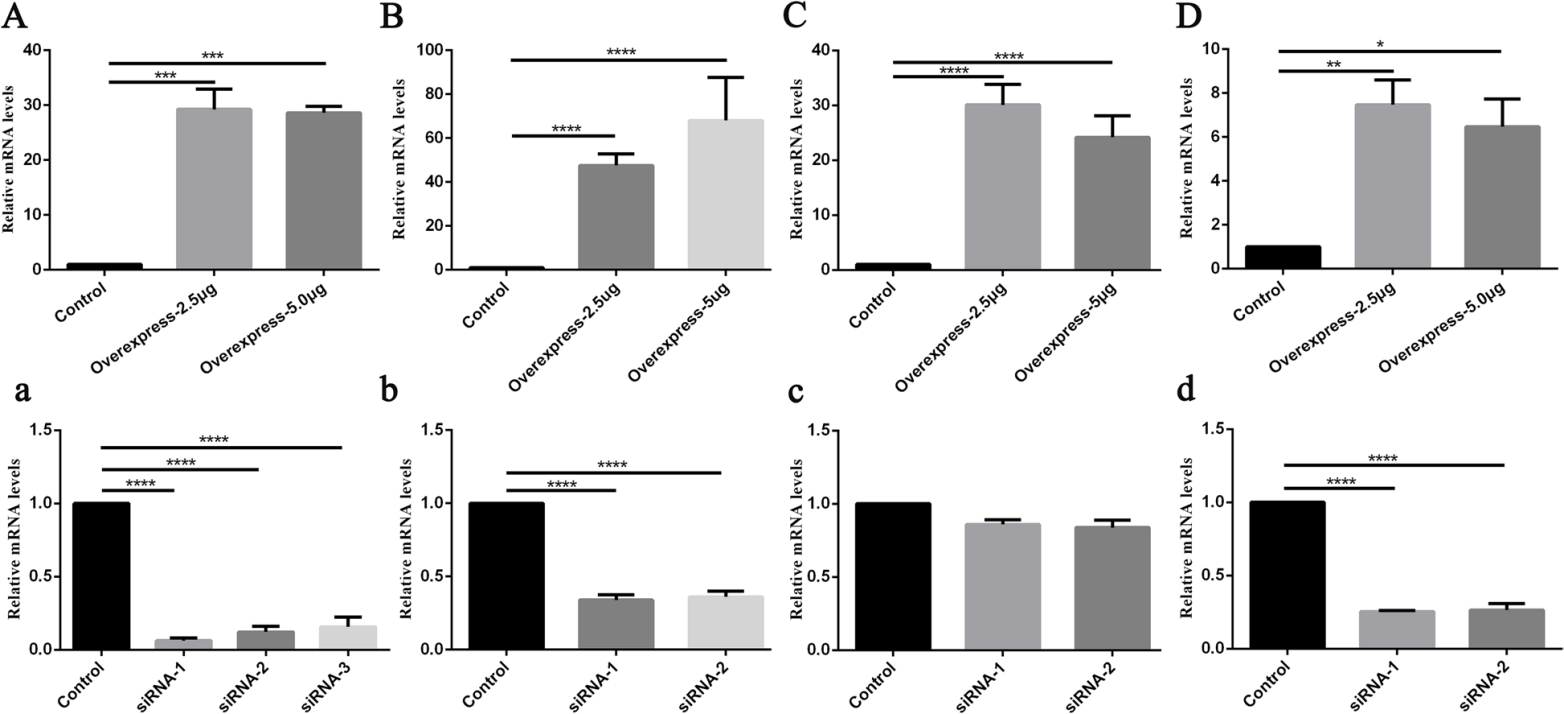
The relative expression level of *FABP4* (**A**, **a**), *GSTT2B* (**B**, **b**), *FOXO6* (**C**, **c**) and *PLA2G4A* (**D**, **d**) in transfected adult GCs. "Control" represents transfection with the empty plasmid; "Overexpress" represents transfection with overexpression vectors (transfected with 2.5 or 5.0 μg of plasmids); "siRNA" represents transfection with siRNAs (the efficiency of 3 different siRNA were examined). The results are expressed as mean ± SEM. * *p* < 0.05, ** *p* < 0.01, *** *p* < 0.001,**** *p* < 0.0001

To further investigate the cellular function of genes, four genes including *FABP4*, *PLA2G4A*, *FOXO6* and *GSTT2B*, were selected for functional examination in cells. We transfected overexpression vectors of these genes into adult GCs and achieved high expression of the target genes in cells (Fig. 6A-D). While, except for *FOXO6*, transfection of siRNAs could lead to significant decrease of expression of the target genes (Fig. 6a-d).

#### The function of FABP4 in GCs

**Fig. 7:**
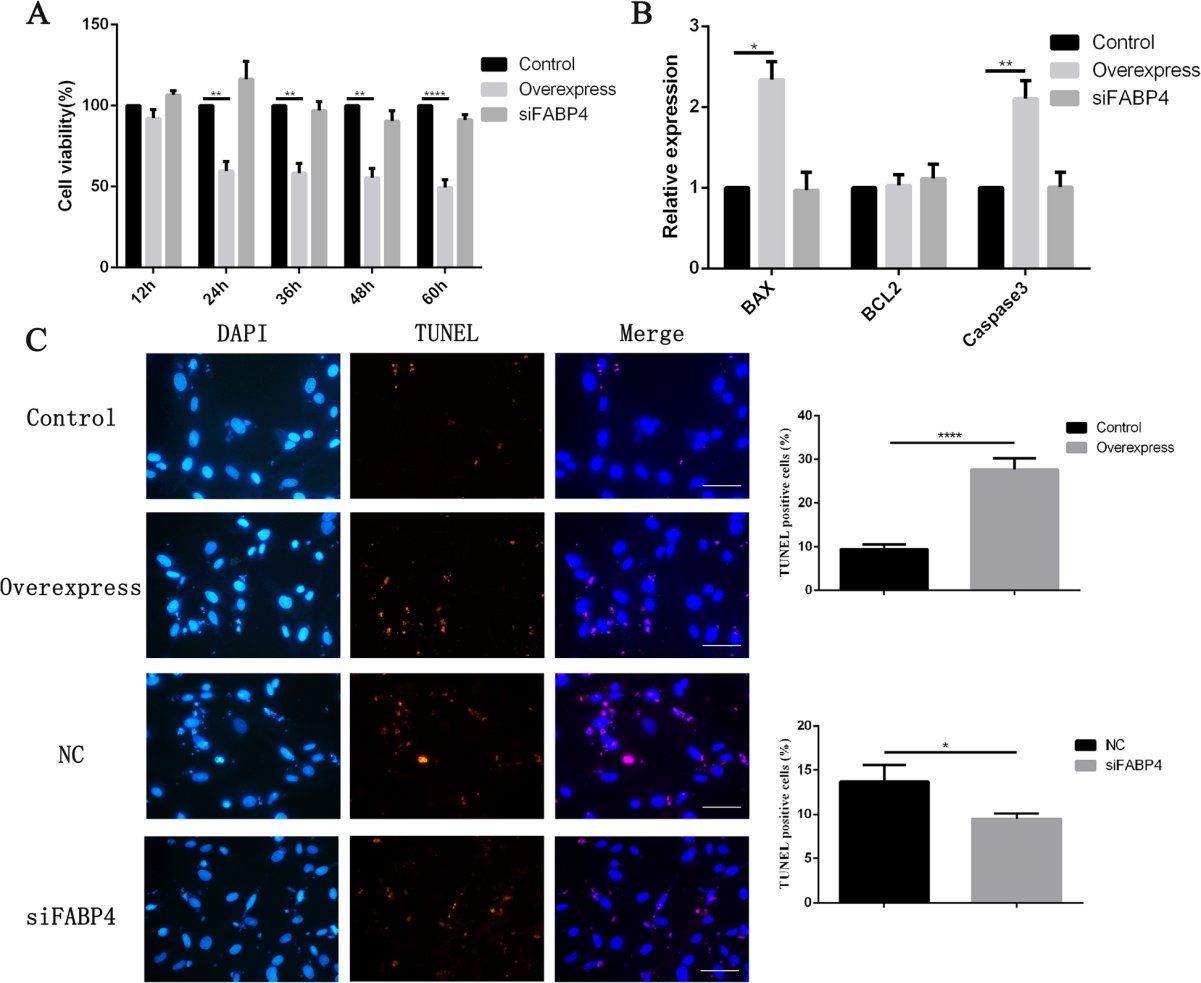
Functional verification of *FABP4* in GCs. **A** The cell viability after overexpression and interference of *FABP4* in adult GCs. The value of control group (0) was set to 100%. **B** The relative expression levels of *BAX*, *BCL2* and *Caspase3* after overexpression and interference of *FABP4* gene in adult GCs. **C** Detection of cell apoptosis by TUNEL. Cells were stained with TUNEL solution (red) and DAPI (blue). Adjustment of colors was applied in entire images to avoid reducing signals on merged images, and representative images are shown. Scale bar, 20 μm. The histogram is the quantification of TUNEL staining. The results are expressed as mean ± SEM. "Control" represents transfection with the empty plasmid; "Overexpress" represents transfection with the *FABP4* overexpression vector; "siFABP4" represents transfection with siRNA; "NC" stands for interference negative control group.* *p* < 0.05, ** *p* < 0.01, *** *p* < 0.001,**** *p* < 0.0001

The effect of *FABP4* on proliferation and apoptosis of GCs from adult ewes was examined. Compared with the control group, the GC viability began to significantly decrease at 24 h after transfection with *FABP4* overexpression vector, and the cell activity at 36, 48 and 60 h after transfection was significantly lower than that of the control group (Fig. 7A). However, interference of *FABP4* had no significant effect on the GC activity.

We then examined the expression of apoptosis-related genes (*BAX*, *BCL2* and *Caspase3*). We found that the relative expression levels of pro-apoptotic gene *BAX* and *Caspase3* were significantly increased in *FABP4* overexpressed GCs from adult ewes, while no significant changes were observed in the expression of anti-apoptotic gene *BCL2*. However, there were no significant differences in expression of *BCL2*, *Caspase3* and *BAX* between the interference and control groups (Fig. 7B). TUNEL assay showed that the proportion of TUNEL-positive GCs was increased in the overexpression group, while the apoptotic ratio was decreased in the interference group (Fig. 7C).

#### The function of PLA2G4A in GCs

**Fig. 8:**
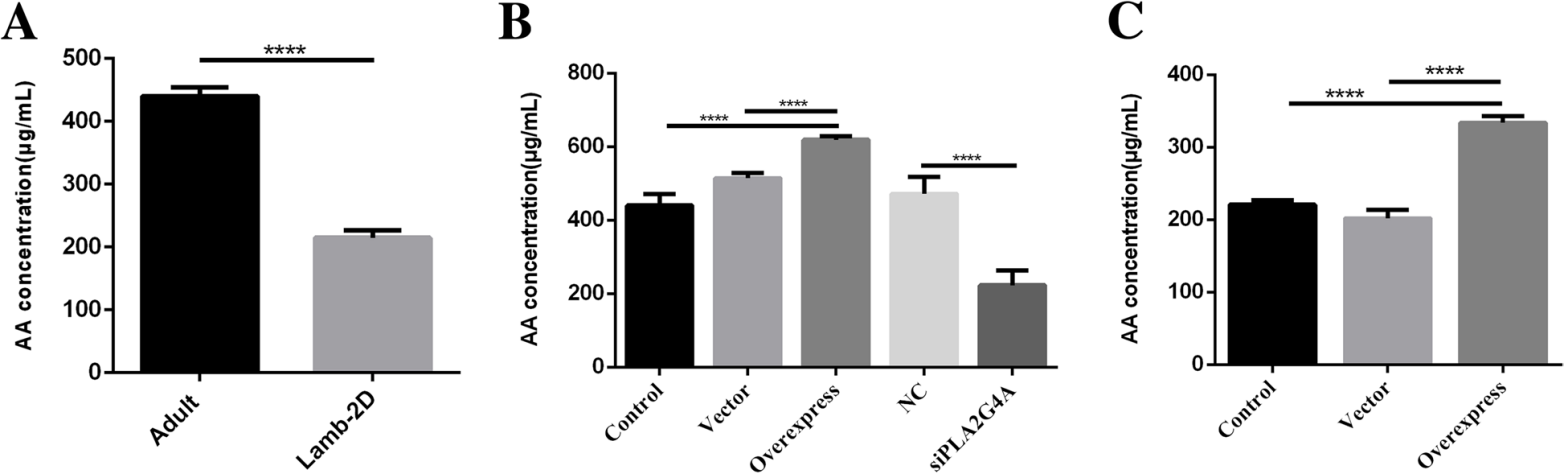
Functional verification of *PLA2G4A* in GCs. The concentration of arachidonic acid (AA) in cell culture medium was measured using a detection kit. **A** The ability of GCs to secrete AA. "Adult" represents GCs from adult ewes, and "Lamb-2D" represents GCs from lambs with 2-day FSH-treatment. **B** Effects of overexpression or interference of *PLA2G4A* in adult GCs on secretion of AA. **C** Effects of overexpression of *PLA2G4A* in lamb GCs on secretion of AA. "Control" represents the untreated cells; "Vector" represents the cells transfected with the empty plasmid, pIRES2-ZsGreen1;"Overexpress" represents the cells transfected with the *PLA2G4A* overexpression vector. "NC" stands for interference negative control group;"siPLA2G4A" represents transfection with siRNA. **** *p* < 0.0001

*PLA2G4A* is a cytosolic phospholipase that can promote the production of arachidonic acid (AA), a substrate of prostaglandin synthesis. We measured the level of arachidonic acid in culture medium of GCs, and found that the AA concentration in medium of lamb GCs was significantly lower than that of adult GCs (Fig. 8A).

Overexpression of *PLA2G4A* in adult GCs significantly increased the content of AA in the medium and interference of *PLA2G4A* decreased the AA level. In lamb GCs (Fig. 8B), overexpression of *PLA2G4A* also resulted in a significant increase of AA levels (Fig. 8C).

#### 23The function of GSTT2B in GCs

**Fig. 9:**
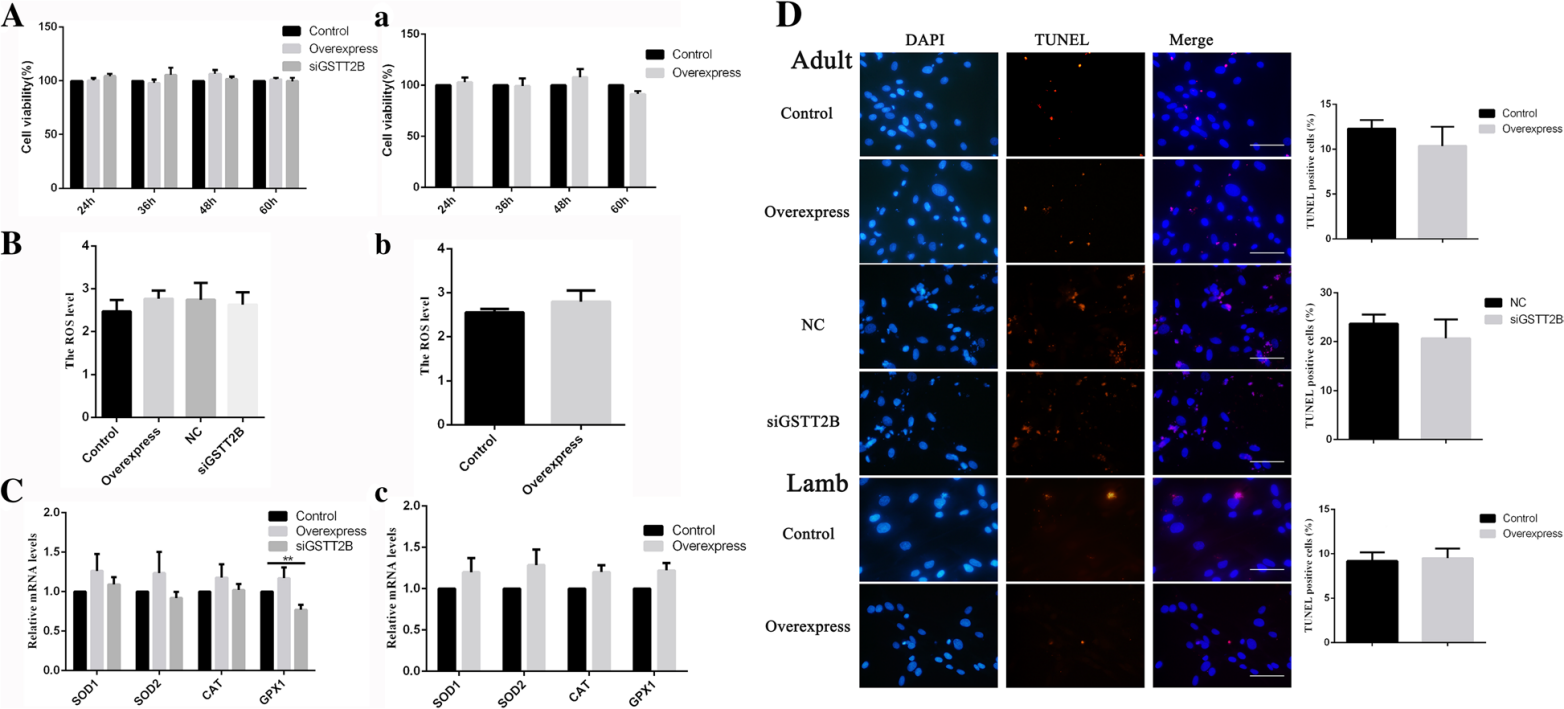
Functional examination of *GSTT2B* in GCs. **A**-**a** The cell viability after overexpression or interference of *GSTT2B* in adult (**A**) and lamb (**a**) GCs. **B**-**b** The cellular ROS levels after overexpression or interference of *GSTT2B* in adult (**B**) and lamb (**b**) GCs. ROS levels were measured using a detection kit and were reflected by fluorescence intensity in each 1 × 10^4^ cells. **C–c** Relative expression levels of antioxidant genes, *SOD1*, *SOD2*, *CAT* and *GPX1* in adult (**C**) and lamb (**c**) GCs after overexpression or interference of *GSTT2B*. **D** Detection of cell apoptosis by TUNEL. Representative images of TUNEL (red) and DAPI (blue) stained cells are shown. Scale bar, 20 μm. The histogram is the quantification of TUNEL staining. The results are expressed as mean ± SEM. "control" represents the transfection with the empty plasmid, pIRES-puro3. "Overexpress" represents the transfection with *GSTT2B* overexpression vector. "NC" stands for interference negative control group; "siGSTT2B" represents the transfection with siRNA

The effects of *GSTT2B* on antioxidation, proliferation and apoptosis of GCs were investigated. The results showed that the expression levels of antioxidant genes *SOD1*, *SOD2*, *CAT* and *GPX1* did not change significantly after *GSTT2B* overexpression in either adult (Fig. 9C) or lamb GCs (Fig. 9c). The expression of *GPX1* was significantly decreased in the interference group (Fig. 9C), while no obvious changes were observed in the expression of *SOD1*, *SOD2* and *CAT* (Fig. 9C and c). We further found that overexpression or interference of *GSTT2B* had no significant effect on the ROS levels (Fig. 9B and b), cell viability (Fig. 9A and a) and apoptosis (Fig. 9D and d) in both adult and lamb GCs.

#### The function of FOXO6 in GCs

**Fig. 10:**
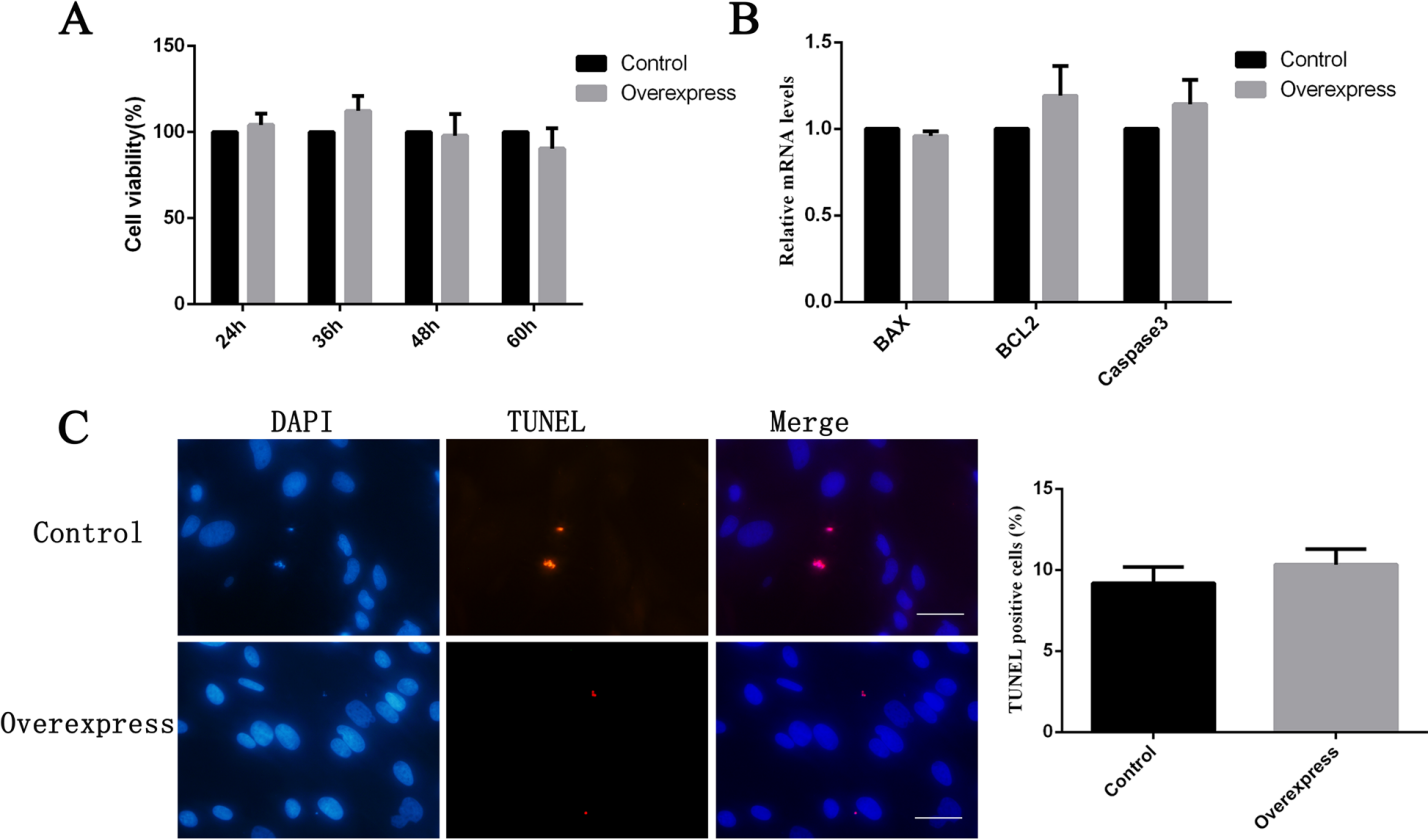
Functional examination of *FOXO6* in GCs. **A** The cell viability of lamb GCs transfected with the *FOXO6* overexpression vector. The value of control group (0) was set to 100%. **B** The relative expression levels of *BAX*, *BCL2* and *Caspase3* in lamb GCs after overexpression of *FOXO6*. **C** Detection of cell apoptosis of lamb GCs. Cells were stained with TUNEL solution (red) and DAPI (blue), and representative cells are shown. Scale bar, 20 μm. The histogram is the quantification of TUNEL staining

*FOXO6* is related to regulation of cell apoptosis and the expression of *FOXO6* was significantly lower in lamb compared to adult GCs. We found that, however, overexpression of *FOXO6* in lamb GCs had no significant influence on cell viability, cell apoptosis and the relative expression levels of *BAX*, *BCL2* and *Caspase3* in cells (Fig. 10A-C). As the siRNAs tested were not efficient for knockdown of *FOXO6* expression, the interference experiment was not done in the study.

## Discussion

Low in vitro developmental ability of prepubertal oocytes suggests that the oocytes undergo insufficient development during growth in vivo. In the sheep JIVET program, the lambs are usually treated with injection of FSH for 2 days prior to oocyte recovery [5, 14]. In this study, we showed that FSH-treatment for 3 days significantly enhanced the development of lamb oocytes when compared to FSH-treatment for 2 days, although it was still not comparable to adult oocytes from the same treatment protocol. Thus, increasing FSH-treatment and extending the in vivo development duration of prepubertal oocytes would be favorable to the improvement of oocyte competence.

It is well known that there is a bidirectional communication between GCs and oocytes during development in follicles [6, 7]. GCs can coordinate the nuclear and cytoplasmic maturation of oocytes [15] and affect the growth and development of follicles [16]. Therefore, analysis of GC characteristics can be used to evaluate the quality of follicles and oocytes.

In this study, we used transcriptomic analysis to investigate the possible functions of GCs in FSH-treated lambs and found dramatic differences in gene expression patterns between lamb and adult GCs. Especially, a total of 405 DEGs were found in lambs with 2-day FSH-treatment vs. adult ewes, which is similar to a previous report claiming 311 DEGs between lamb and adult GCs [9]. However, the number of DEGs decreased to 159 in lambs with 3-day FSH-treatment vs. adult ewes. The numbers of significantly enriched (q-value ≤ 0.05) GO terms and pathways from A vs. C were significantly less than that of A vs. B, which indicated that lengthening FSH treatment time probably made the follicular pattern of lambs more similar to adult ewes. Thus, compared to FSH treatment for 2 days, lambs with FSH-treatment for 3 days had been probably improved on some biological functions, such as cellular response to transforming growth factor beta stimulus, regulation of cell proliferation and extracellular vesicular exosome, etc.

We further investigated the DEGs by pairwise comparative analysis of the three groups and found 101 common DEGs shared with both 2-day and 3-day FSH-treatment of lambs vs. adults. These DEGs may represent the intrinsic differences between GCs of lamb and adult sheep, regardless of extending FSH-treatment to 3 days. Analysis of KEGG pathway combined with GO terms indicated that these common DEGs were enriched in biological processes such as cell adhesion, extracellular matrix organization, regulation of cell proliferation and so on. These DEGs were significantly enriched in some pathways including adherens junction, ECM-receptor interaction, cell adhesion molecules and PI3K-Akt pathway and so on. Among them, adherens junction (AJs) is the most common type of intercellular adhesion, which is pivotal for maintaining tissue structure and cell polarity, and can restrict the movement and proliferation of cells [17]. Cell adhesion molecule (CAMs) is a glycoprotein expressed on the cell surface, which plays an important role in a series of biological processes, including hemostasis, immune response, inflammation, embryonic development and neuronal tissue development [18, 19]. In ECM-receptor interaction signaling pathway, extracellular matrix is composed of complex mixtures of structural and functional macromolecules, including glycoproteins, proteoglycans, aminoglycans and so on. It plays a major role in the morphogenesis of tissues and organs and in maintaining the structure and function of cells and tissues [20]. The interaction between cells and extracellular matrix leads to direct or indirect control of cellular activities, such as adhesion, migration, differentiation, proliferation and apoptosis [21]. In addition, extracellular matrix is also involved in the process of cumulus expansion and plays a role in oocyte maturation [22]. PI3K-Akt signal transduction pathway is activated by Akt phosphorylation and participated in the regulation of basic cellular functions, such as the transcription, translation, proliferation and growth [23].

In order to further clarify the function mechanism of the differences in gene expression between lamb and adult GCs, we drew the PPI networks. The results showed that *CD83* was the core gene of three PPI networks. The expression of *CD83* was up-regulated in lamb GCs. Previous studies have shown that CD83 is a member of the immunoglobulin (Ig) superfamily, which is elevated in the serum of patients with autoimmune disease and hematological malignancies and has an immune suppressive function [24]. In addition, Liu et al. proposed that ovulation involved the expression and function of molecules that exert potent roles in innate immune responses, and granulosa cells appeared to play immuno-protective-like functions for the ovulated oocytes [25]. Another report found that the possible initiation of early follicular atresia in small antral follicles during the follicular phase had an interaction with the presence of immune cells [26]. Our results suggest that up-regulation of *CD83* in lamb GCs may have an adverse effect on the immune system and development of oocytes.

Enrichment of function analysis revealed same and different enrichment of GO terms between A vs. B and A vs. C. The candidate genes from PPI network of A vs. B were enriched in endothelial cell migration, fatty acid transport, Rho GTPase binding and collagen-containing extracellular matrix, and so on. Rho GTPase binding is participated indirectly in oocyte maturation, blastocyst formation and stem cell development by regulated Rho-associated coiled-coil-containing protein (ROCKs) [27]. Endothelial cell migration is essential for angiogenesis [28]. Angiogenesis is participated in the follicular development and ovulation [29], and thus there is a certain connection between the two. Extracellular matrix (ECM) constitutes the follicular basal lamina and is responsible for aggregation of granulosa cells, and it is vital for supporting ovarian follicle growth and maintaining its proper function [30]. In this study, we found some DEGs encoding ECM-related proteins in collagen-containing extracellular matrix (A vs. B) and complex of collagen trimmers (A vs. C), such as collagens *COL15A1*, *FBN1* (A vs. B) and *COL4A5*, *COL4A6*, *COL5A2* (A vs. C) [31]. Therefore, the enrichment of GO terms was related to the differences in follicular development and ovulation between lamb and adult ewes.

We verified the expression of 19 DEGs that are implicated in follicle and oocyte development by qRT-PCR. Inhibin B (*INHBE*, also known as activin E) is expressed in GCs of early antral follicles and dominant follicles, which promotes the development of single dominant follicle by inhibiting the secretion of pituitary FSH in follicular phase [32]. Follistatin (*FST*) is a binding protein of activins. The activin-follistatin system controls the growth and differentiation of antral follicles by affecting the production of gonadotropin receptors and steroids [33]. As a follicle develops to a dominant follicle, activin secretion is decreased, while inhibin and follistatin production are increased [34]. In our results, *INHBE* and *FST* were expressed lowly in lambs GCs, which may hinder the selection of dominant follicles and permit a large number of lamb antral follicles to grow in response to FSH stimulation.

*ERBB4* is one of the four receptors in mammalian EGFR system [35]. Studies have demonstrated that EGF-like factors and EGFR signaling pathway played vital roles in the regulation of oocyte meiosis resumption, the expansion of cumulus cells and ovulation [36, 37]. Prevot et al. found that there was a significant delay in puberty in *ERBB4* mutant mice, and their reproductive function was also impaired in adulthood [38]. In view of this, ERBB4 may play a certain role in the reproductive process, and its low expression in lamb GCs may not facilitate the activation of the EGFR signaling pathway, thus reflecting some developmental defects of lamb oocytes.

In agreement to a previous report [9], we found that *HTRA3* was differently expressed between adult and lamb GCs. *HTRA3* is a member of the HtrA family of serine proteases and plays an important role in ovarian development, GC differentiation and luteinization [39]. It was reported that the expression level of *HTRA3* was significantly increased in luteinized GCs [40]. In this study, we found that the expression of *HTRA3* in lamb GCs is significantly higher than that in adult GCs. This implies that lamb GCs may undergo luteinization, which is not favorable to the full development of follicles and oocytes.

*ESR2*, *TGFβ1*, *TNFAIP6*, *PTGS2* and *AFP* are related to ovulation. As a receptor of estrogen, *ESR2* is critical for full development and maturation of follicles and oocytes [41]. *TGFβ1* can induce *PTGS2* expression and prostaglandin E2 (PGE2) production in GCs through Smad signaling pathway, which further promotes oocyte maturation and ovulation [42]. *TNFAIP6* specifically expresses in mucilaginous liquefaction cumulus-oocyte complexes (COCs) [43], which is indispensable for the formation of extracellular matrix in COCs during ovulation [44]. Prostaglandin is involved in ovulation, and *PTGS2* plays a key role in synthesis of prostaglandins [45]. *AFP* is a serum glycoprotein. Female mice with *AFP* knockout were unable to ovulate due to hypothalamic dysfunction [46]. The expression of these genes was significantly lower in lamb GCs than adult GCs, which may be the reasons that lamb follicles rarely ovulate.

*FBN1* is a glycoprotein and is involved in regulating the activity of *TGFβ*. When *FBN1* is silenced, the proliferation of cumulus cells is significantly increased and the apoptosis of cumulus cells is prevented [47]. The high expression of *FBN1* in lamb GCs may inhibit the proliferation of GCs and affect the normal secretory and metabolic process of GCs.

*SCUBE2* is a new type of vascular growth factor receptor 2 co-receptor and can enhance the signal transduction induced by vascular endothelial growth factor (VEGF) during angiogenesis, thus promoting angiogenesis [48]. *ANGPT1* and *ANGPT2* are participated in maturation and stabilization of blood vessels via tyrosine kinase receptors [49, 50]. *ID3* is involved in the control of cell cycle and cell fate, and may also play an important role in angiogenesis [51]. Fraser et al. suggested that angiogenesis plays a critical role in follicular development, and interference with the process of angiogenesis can inhibit follicular development or prevent ovulation [52]. Low expression of *ANGPT1*, *ANGPT2* and *SCUBE2* in lamb GCs may hinder follicular maturation and ovulation by inhibiting follicular angiogenesis.

In this study, we examined the functions of four genes, *FABP4*, *PLA2G4A*, *GSTT2B* and *FOXO6*, in ovine GCs. *FABP4* is expressed mainly in adipocytes and has been widely studied in obesity metabolic syndrome and cardiovascular disease [53]. Van et al. observed that the ultrastructural characteristics of apoptotic GCs in bovine ovary was similar to those of *FABP4* positive cells [54]. High expression of *FABP4* was observed in the GCs of atretic follicles in mice [55]. It was found that *FABP4* is closely related to the occurrence of polycystic ovary syndrome [56]. In this study, we found that *FABP4* was highly expressed in GCs of lambs and demonstrated that overexpression of *FABP4* promoted the apoptosis of GCs. *FABP4* may play its role through PPARγ signal pathway [55]. It is also suggested that the promotion of apoptosis by *FABP4* may be related to the up-regulation of *TNFAIP6* and down-regulation of TGF-α [57]. These may be the reasons that *FABP4* promotes apoptosis, but other mechanisms may also exist and need to be further investigated.

*PLA2G4A* is a cytosolic phospholipase that can promote the production of arachidonic acid (AA), a substrate of prostaglandin synthesis [58]. Boruszewska et al. suggested that PGE2 could facilitate successful oocyte maturation and oocyte survival in the cow [59]. In this study, the expression of *PLA2G4A* in GCs of adult ewes and 3-day FSH-treated lambs were higher than that of 2-day FSH-treated lambs. Overexpression and RNAi experiments demonstrated that *PLA2G4A* had an important effect on the ability of GCs to secrete AA. AA is a substrate for the synthesis of prostaglandin E2 that regulates the cumulus cells expansion [60]. Thus, the defective development of oocytes and follicles in lambs may be partially due to the low concentration of prostaglandin E2, and prolonged FSH treatment of lambs would be beneficial to the *PLA2G4A* expression and prostaglandin E2 synthesis, thereby promoting the oocyte maturation and development. In addition, Sapieha et al. believed that AA, as an unsaturated fatty acid, promotes angiogenesis [61]. Therefore, the low concentration of prostaglandin E2 in lamb follicles may reduce the formation of mature blood vessels on follicles, and thereby affect the oocyte maturation and follicular ovulation.

GSTs are involved in glutathione-dependent oxidation resistance [62]. *GSTT2B* is a member of the GSTs family, but its function is little known. It may act as an antioxidant, promoting detoxification of reactive oxygen species (ROS). Our results suggested that *GSTT2B* was expressed lowly in lamb GCs. Therefore, we speculated that lamb GCs may have a lower antioxidant capacity related to *GSTT2B* deficiency. However, we found that overexpression of or interference with this gene in lamb and adult GCs, had no significant roles in antioxidation and apoptosis in cells.

FOXO factors are important regulators of cell cycle, apoptosis, DNA repair, antioxidant stress and cell lifespan [63, 64]. *FOXO6* is a member of the FOXO family and is a vital regulator of insulin or liver glucose metabolism [65]. Knockdown of *FOXO6* in colorectal cancer cells inhibited the cell proliferation, migration, invasion and glycolysis [66]. So far, there is no report of *FOXO6* in ovine GCs. Our study showed that the expression of *FOXO6* was significantly decreased in GCs of lamb treated with FSH for 2 and 3 days. We thought that *FOXO6* might be a critical gene in regard to the difference in GC functions between lambs and adult ewes, and it may be related to cell proliferation. However, *FOXO6* had no effect on cell proliferation and apoptosis in our assays. The role of *FOXO6* in GCs needs further investigation.

## Conclusions

In this study, we show that there were notable differences in transcriptional patterns of GCs between lambs and adult ewes, and a number of differently expressed gene were identified between the two groups of females, which partially explains the possible reasons for the developmental defects in lamb follicles and oocytes. Although the real actions of these differently expressed genes need to be further investigated, two genes, *FABP4* and *PLA2G4A*, have been shown to have a function in granulosa cells like apoptosis and arachidonic acid synthesis. Finally, we demonstrate that prolonging the FSH-treatment of lambs would be favorable to the GC functions and oocyte development. Our study provides important data for further understanding the mechanism of follicular development in prepubertal animals and improving their oocyte developmental competence.

## Materials and methods

### Animals, hormonal treatment and sample collection

The procedure of all animal experiments was in accordance with the animal care policies of China Agricultural University and was approved by the Animal Ethics Committee at the university. During handling of animals, all efforts were made to minimize the pain and suffering experience of animals.

Dorper breed female lambs and adult ewes with good health were provided by Inner Mongolia Sino Sheep Technology Co. Ltd and kept in a farm of this company located at Wulanhua town, Wulanchabu, Inner Mongolia, China(41^。^33′N, 111^。^38′E). All animals, including lambs with their mothers, were house-fed under conventional management conditions. Hormonal treatment of animals and surgery collection of samples were performed in early autumn (average temperature was around 19.5℃). A total of 12 experimental lambs (4–6 wk old) were randomly allocated to two treatment groups and each lamb received 180 IU follicle-stimulating hormone (FSH) (Sansheng Pharmaceutical, Ningbo, China) in total, including 4 × 45 IU (2-day group; *n* = 6) or 6 × 30 IU (3-day group; *n* = 6), given at approximately 12 h intervals. At the time of the first FSH injection, lambs were simultaneously injected with 400 IU equine chorionic gonadotropin (eCG; Sansheng). Adult ewes were also used as oocyte donors for embryo in vitro production (IVP) and this technology has been routinely applied in sheep breeding in the company, Inner Mongolia Sino Sheep Technology. In parallel with the experiments on lambs, a total of 100 adult ewes (3–4 y old) were used to IVP program during that period. Thus, samples from these ewes were collected for comparisons with lambs. The ewes were implemented an intravaginal CIDR (InterAg, New Zealand) at any days of the cycle and 12 d later they received 6 × 30 IU injection of FSH with an interval of 12 h for each injection. All animals were subjected to one cycle of hormonal treatment and used only once in the experiments.

Oocytes were recovered from lambs and ewes approximately 12 h after the last FSH injection as described previously [67]. The animals were induced to general anesthesia by intramuscular injection of anesthetic (Su-Mian-Xin II, Institute of Military Veterinary, Changchun, China; injection dosage: 5 μl/kg body weight) and ovaries were exposed by mid-ventral laparotomy by surgery. The animals were carefully monitored for a week after surgery, and then maintained under normal conditions. Follicles ≥ 2 mm in diameter on ovarian surface were punctured and aspirated using a 10 G needle disposable syringe. The cumulus-oocyte complexes (COCs) were picked under a stereo microscope and used for in vitro maturation. The remaining GCs were collected and cultured in DMEM/F12 supplemented with 10% fetal bovine serum (FBS) for 2–3 d. When the cell cultures reached 80% of confluence, the cells were harvested and stored at -80 °C until RNA extraction.

### Oocyte in vitro maturation (IVM) and fertilization (IVF)

The procedure of oocyte IVM/IVF was same as described previously [67]. Briefly, COCs were cultured at 38.5 C for 24 h in a humidified atmosphere of 5% CO_2_ in TCM199 supplemented with 20% (v:v) estrous sheep serum (ESS), 10 μg/mL FSH (Folltropin-V; Bioniche Inc., Belleville, Ont., Canada), 10 μg/mL LH (Bioniche Inc) and 1 μg/mL 17β-estradiol. After maturation, the oocytes were fertilized in vitro using frozen-thawed sperm and incubated in IVF medium, synthetic oviduct fluid (SOF) supplemented with 2% ESS. After 20 h, the presumptive fertilized oocytes were cultured in SOF medium containing 8 mg/mL fatty acid-free bovine serum albumin, 1% (v:v) essential amino acids and 2% (v:v) non-essential amino acids, at 38.5 C in a humidified atmosphere of 5% CO_2_, 7% O_2_ and 88% N_2_. The cleavage of fertilized oocytes and blastocyst development of embryos were evaluated after 2 d and 7 d of culture, respectively. All embryos generated in the experiments, including 945 2-cell embryos and 209 blastocysts, were transferred into recipient ewes for producing offspring. The data on preimplantation development of embryos was presented in this study (detailed in Table 1).

### RNA-seq and identification of differentially expressed genes

For Illumina sequencing, total RNAs from GCs were extracted by Trizol Reagent (Tiangen, Beijing, China). The GC samples were from three groups of animals, including adult ewes (A), lambs with FSH-treatment for 2 days (B) and for 3 days (C). Each group consisted of 3 biological replicates and each replicate contained a mixed GC sample from 2 individual animals. The quality of total RNAs was assessed using Agilent 2100 Bioanalyzer (Agilent Technologies, Palo Alto, CA). After reverse transcription, cDNA libraries were constructed and sequenced by using Illumina HiSeq™ 2000. After nine sets of raw reads were obtained, the data were deposited by removing the contaminated, datper and low-quality reads, and all reads were uniquely mapped onto the ovine genome (https://www.ncbi.nlm.nih.gov/genome/?term=Ovis%20Aries).

For RNA-seq analysis, we used the Python-based toolkit HTseq to efficiently and accurately mapped reads to genes, and finally used the FPKM (fragment per kilo-bases per million mapped reads) method to calculate the expression level of each gene. Comparisons of FPKM in three groups (A vs. B, A vs. C and B vs. C) were performed, and genes with q-value ≤ 0.01and |log2FC|> 1 were considered as significant differently expressed genes (DEGs) for subsequent analysis.

### Data analysis

We used Gene Ontology (GO) enrichment terms for the functional categories of DEGs. GO has a total of three ontologies, which describe the molecular function of genes, cellular components and biological processes. Then GO enrichment analysis of differentially expressed transcripts was implemented using GO-seq software package. All DEGs were mapped to the Kyoto Encyclopedia of Genes and Genomes (KEGG) (http://www.genome.jp/kegg) to further investigate their functions. GO terms and KEGG pathway with corrected p-values less than 0.05 were considered significantly enriched. The data sets of protein–protein interactions (PPIs) were obtained from the online data-base STRING 11.0 (http://www.string-db.org/) and by a PPI prediction method called domain-domain interaction (DDI) that is based on the conservatism of the domain. After getting the PPIs, we made full use of Cytoscape 3.8.2 to show the PPIs and utilized the Clue-GO to cluster the GO terms and KEGG pathways.

### Validation by quantitative reverse transcription PCR (qRT-PCR)

Purified total RNA of GCs was used as a template for cDNA synthesis by using FastKing gDNA Dispelling RT SuperMix (Tiangen, China). The real-time quantitative PCR was performed using the SuperReal PreMix Plus (Tiangen) in an ABI PRISM 7500 System (Applied Biosystems, USA). The 2^‐ΔΔCT^ method was used to determine the gene expression level. All primers were designed using Primer 5.0 (supplementary Table S5). All samples were analyzed in triplicates and all experiments were repeated for three times at least.

### Overexpression and interference of target genes in GCs

Coding sequences (CDS) of specific target genes were cloned from GC cDNAs. Accession number of the genes and designed primers are shown in supplementary Table S5. The CDS were constructed into eukaryotic expression vectors, pIRES2-ZsGreen1or pIRES-puro3 (Clontech, USA). The small interfering RNAs (siRNAs) targeting genes and their control siRNA (si-con) were designed and synthesized by Sangon Biotech (Shanghai, China). The overexpression vectors or siRNAs were transfected into cultured GCs using Lipofectamine 3000 (Invitrogen, Carlsbad, CA) according to the manufacturer instructions.

### Assessment of cell viability

The CCK-8 assay was used to assess the viability of transfected cells. The cells were seeded in 96-well plates and incubated at 37 °C. The indices of cell proliferation were measured using a CCK-8 kit (Beyotime Institute of Biotechnology, China) at 12, 24, 36, 48, and 60 h after transfection. Then 10 μL CCK-8 solution was added into the well and incubated for 1 h at 37 °C. The optical density was measured using a microplate analyzer and the reference wavelength was set at 650 nm and the detection wavelength was set at 450 nm.

### TUNEL assays

The TUNEL was used for detecting DNA breaks by using One Step TUNEL Apoptosis Assay Kit, red fluorescence (Beyotime). Cell slides from different experimental groups were fixed with 4% paraformaldehyde at room temperature for 30 min and then treated with 0.3% Triton X-100 in PBS for 5 min. After that, 50 μL TUNEL assay solution was dripped to cell slides, and the slides were incubated at 37 °C for 60 min. After cells were counterstained with DAPI, the slides were observed under a Qlympus BX51 epifluorescence microscope equipped with a DP72 microscope digital camera (Olympus Corporation, Tokyo, Japan), and the fluorescent images were acquired with Image-Pro Plus 6.0 software (Media Cybemetics, MD, USA). Six fields were selected randomly from each coverslip for observation and more than 200 cells from each field were recorded. The exposure length for catching each type of fluorescence was kept constant. After subtracting the background of images, the intensities of fluorescence in cells were quantified using Image-J 1.45 software (National Institutes of Health, USA). Apoptosis ratio was calculated as the number of apoptotic cells / total cell numbers.

### Detection of arachidonic acid concentration

Sheep arachidonic acid (AA) ELISA detection kit (Beijing Shijichuangxiang Biotechnology, Beijing, China) was used to detect the concentration of AA in the medium of GCs. The cell culture medium was collected by centrifuge at 1000 rpm for 10 min. Antibodies against AA (100 μL) labeled with horseradish peroxidase (HRP) were added to holes of Elisa Plate contained standard sample and samples except the blank, and they were incubated at 37 °C for 60 min. Then, the liquid was removed and the substrate of A and B were added to the hole of Elisa Plate and incubated for 15 min in dark. Finally, the terminated solution was added to the hole, and optical density was determined immediately at 450 nm by using a microplate analyzer.

### Intracellular ROS measurement

A reactive oxygen species detection kit (Beyotime) was used to detect the levels of ROS in GCs according to the instruction of manufacture. DCFH-DA was added to the cell culture dish and incubated at 37 °C for 20 min. Then the cells were harvested and the number of cells was counted by using the cell count plate. The fluorescence intensity from 1 ml cell suspension was detected by using the fluorescence spectrophotometer, and the fluorescence intensity of each 10,000 cells was calculated.

### Statistical analysis

Analysis of data were performed using SPSS 18.0 software. Homogeneity of variances was analyzed by Levene’s test and normality of the data was tested using the Kolmogorov–Smirnov test. Student *t*-test or one-way ANOVA test with post hoc by least significance difference test (LSD) was used to analyze the data that were normally distributed, and Mann–Whitney U test was used to analyze the data that were not normally distributed. All data are expressed as mean ± standard error. The graphs, transcriptome map and PPI networks were drawn using GraphPad Prism 5.0 software, R × 64 3.6.2 software package and Cytoscape 3.8.2, respectively.

## Supplementary Information

**Additional file 1:** **Table S1.** The results of original data filtering.

**Additional file 2:** **Table S2.** The gene expression of all samples and DEGs between groups.

**Additional file 3:** **Table S3.** Go and pathway enrichment of DEGs.

**Additional file 4:** **Table S4.** The data sets of protein–protein interactions.

**Additional file 5:** **Table S5.** Sequences for primers.

## Data Availability

The datasets generated during the current study are available in the NCBI Sequence Read Archive (SRA), http://www.ncbi.nlm.nih.gov/bioproject/PRJNA753660.

## Abbreviations

AA: Arachidonic acid
AFP: Alpha fetal albumin
ANGPT1: Angiopoietin 1
ANGPT2: Angiopoietin 2
BAX: BCL2 associated X, apoptosis regulator
BCL2: BCL2 apoptosis regulator
BP: Biological process
CAT: Catalase
CC: Cellular component
CD83: CD83 molecule
CDS: Coding sequences
COL4A5: Collagen type IV alpha 5 chain
COL4A6: Collagen type IV alpha 6 chain
COL5A2: Collagen type V alpha 2 chain
CL15A1: Collagen type XV alpha 1 chain
COCs: Cumulus-oocyte complexes
DDI: Domain-domain interaction
DEGs: Differently expressed genes
ECM: Extracellular matrix
ERBB4: Erb-b2 receptor tyrosine kinase 4
ESS: Estrous sheep serum
ESR2: Estrogen receptor 2
FABP4: Fatty acid binding protein 4
FBN1: Fibrillin 1
FF: Follicular fluid
FOXO6: Forkhead boxO6
FSH: Follicle-stimulating hormone
FPKM: Fragment per kilo-bases per million mapped reads
FST: Follistatin
GCs: Granulosa cells
GO: Gene Ontology
GPX1: Glutathione peroxidase 1
GSTT2B: Glutathione S-transferase β-1
KEGG: Kyoto Encyclopedia of Genes and Genomes
HTRA3: HRP: Horseradish peroxidase
HTRA3: HtrA serine peptidase 3
ID3: Inhibitor of DNA binding 3
INHBE: Inhibin β E subunit
IVF: In vitro fertilization
IVM: In vitro maturation
JIVET: Juvenile in vitro embryo transfer
MF: Molecular function
PGE2: Prostaglandin E2
PLA2G4A: Cytoplasmic phospholipase A2
PPIs: Protein–protein interactions
PTGS2: Prostaglandin-endoperoxide synthase 2
ROCKs: Rho-associated coiled-coil-containing protein
SCUBE2: Signal peptide-CUB-EGF domain protein 2
siRNAs: Small interfering RNAs
SOD1: Superoxide dismutase 1
SOD2: Superoxide dismutase 2
SOF: Synthetic oviduct fluid
TGFβ1: Transforming growth factor β 1
TNFAIP6: Tumor necrosis factor α inductive protein
VEGF: Vascular growth factor
