# Supplementary material for: Transcriptomic comparison of ovarian granulosa cells between adult sheep and prepubertal lambs
Source: BMC Genomics. 2022 Feb 21;23:151. doi: 10.1186/s12864-022-08379-x (PMC8862527; doi:10.1186/s12864-022-08379-x)
Supplement: Supplementary file 5 — Additional file 5: Table S5. Sequences for primers. [file 12864_2022_8379_MOESM5_ESM.pdf]

Supplementary Table 5. Sequences for primers.

Accession number	Genes	Sequence (5'-3')	Length (bp)
443005	*GAPDH*	Forward: TGATGACATCAAGAAGGTGGTG<br>Reverse: TCCTTGGAGGCCATGTAGGCCAT	240
443066	*ESR2*	Forward: CCAGCCCTGTTTCTAGTC<br>Reverse: CGTCGGTTCTTATCTATGGT	173
101103044	*ERBB4*	Forward: ATGGCGGCGTCTATGT<br>Reverse: CCCGAGCTACCATTTGTTGA	104
443323	*FST*	Forward: CCTGTCGGGATGTTTTCTGT<br>Reverse: GTAGGCAGGTAGCTTTTCTC	160
100415775	*ID3*	Forward: ATCTTCCCATCCAGACAGCCG<br>Reverse: AAGTCAAGTGGGCAGGGCAA	196
101106006	*TGFβ-1*	Forward: GAGCCCTGGACACCCAACTAC<br>Reverse: CCAGGACCTTGCTGTACTGTG	189
101111472	*PLA2G4A*	Forward: CAGAGGAGATTAACGAAGA<br>Reverse: CCTCCATAAAGACTCAACA	101
101119185	*TNFAIP6*	Forward: GATACTGTGGAGATGACCTT<br>Reverse: GAGTGGATTTGGATAGAGGA	141
101121588	*INHBE*	Forward: CTTCCATTCCGCTGTC<br>Reverse: TGCCATCACGGTCAAGGTAG	99
101109620	*FBN1*	Forward: AACACGCAGGGCTCTTACAC<br>Reverse: ATCAATGTCTCGGCACTCT	81
101103646	*AFP*	Forward: CCCTGGCAAAACGAAGC<br>Reverse: ACTGAGATGGCAACAAACGG	167
101105743	*LAMB1*	Forward: TTAGCCCTGTGCGGAGTT<br>Reverse: TACAGTAAGGCTCGGGTTTG	163
497271	*ANGPT1*	Forward: AAGGAGCGAGTTTTGCGAGA<br>Reverse: TGCTGCACCCTATGTGAGTC	108
497273	*ANGPT2*	Forward: AACCCTTCATACTCGCTTGC	179

		Reverse: CGCTGAATAACCGTCCACC	
101102010	*SCUBE2*	Forward: GTGGACGAGTGCCTGGAGAA<br>Reverse: GCCTCCTTGCAGATGTGACT	188
443460	*PTGS2*	Forward: TTGATTGAGAGTCCGCCAAC<br>Reverse: TTTCCTCCCTTTCACACCCA	141
100137067	*FABP4*	Forward: AGAAGTGGGTGTGGGCTTTG<br>Reverse: CTGGCCCAATTTGAAGGACA	142
443031	*CASPASE3*	Forward: CCGAAAGGTAGCGACAG<br>Reverse: GTTTGGTCACTTGGCATACA	163
443059	*BAX*	Forward: TCTACTTTGCCAGCAAACTGGTGC<br>Reverse: AAGGAAGTCCAATGTCCAGCCCAT	109
101119602	*BCL2*	Forward: TTCGCCGAGATGTCCAGTCA<br>Reverse: TCCGAACTCAAAGAAGGCCACGAT	125
100307035	*CAT*	Forward: CTTCAACAGTGCCAGCGAT<br>Reverse: GTTCTCACACAGGCGTTTC	94
100820742	*GPX1*	Forward: TGCGAGGTGAATGGCGAGAA<br>Reverse: GAGACGTCGTTGCGGCACAC	140
100270717	*SOD1*	Forward: AAGGGAGATAAAGTCGTCGTA<br>Reverse: TTCACATTGCCCAGGTCTC	191
780457	*SOD2*	Forward: CCGTCAGCCTTACACCAAGT<br>Reverse: CAAGCCACGCTCAGAAACAC	112
101123434	*FOXO6*	Forward: CGACCTCATCACCAAAGCCA<br>Reverse: ACGGACCATCCAGTCGTAG	79
101111664	*GSTT2B*	Forward: GCCCGCAAGTATAAGGTCCC<br>Reverse: TCACCCAGGAAAACAGGAA	155
101111664	*GSTT2B* (CDS)	Forward: GAATTCATGGGCCTGGAGCTCTACCTGGACCTGCTGT<br>Reverse: GGATCCTTACTGGATCATGGCCAGCACTTTG	723
100137067	*FABP4* (CDS)	Forward: GAATTCATGTGTGATGCATTTGTAGG<br>Reverse: GGATCCTTATGCTCTCTCGTAAACT	399

101111472	*PLA2GA4*(CDS)	Forward: GAATTCATGTCATTTATAGATCCTTATC<br>Reverse: GGATCCTTATGCTGTGGGTTTGCT	2262
101123434	*FOXO6* (CDS)	Forward: GAATTCATGGCTGCGAAGCTGCGAGCGCATCA<br>Reverse: GGATCCTCAGCCTGGCACCCAGCTCTGGTTG	1710
